# Functional characterization of *BrPHD58*, an Alfin-like PHD finger protein from *Brassica rapa*, reveals its negative role in salt stress tolerance in *Arabidopsis thaliana*

**DOI:** 10.3389/fpls.2026.1749944

**Published:** 2026-01-28

**Authors:** Intikhab Alam, Khadija Batool, Hantao Ge, Hakim Manghwar, Naveed Ur Rehman, Fang Qiao, Hui-Cong Wang

**Affiliations:** 1 Key Laboratory of Biology and Genetic Improvement of Horticultural Crops-South China College of Horticulture, South China Agricultural University, Guangzhou, China; 2 School of Food and Drugs, Shenzhen Polytechnic University, Shenzhen, China; 3 Key Laboratory of Ministry of Education for Genetics, Breeding and Multiple Utilization of Crops, College of Crop Science, Fujian Agriculture and Forestry University, Fuzhou, China; 4 Lushan Botanical Garden, Jiangxi Province and Chinese Academy of Sciences, Jiujiang, China

**Keywords:** *Brassica rapa*, *BrPHD58*, PHD finger protein, salt stress, salt-responsive genes

## Abstract

The plant homeodomain (PHD) finger constitutes a subgroup of transcription factors that contribute to the coordination of plant growth, morphogenesis, and adaptation to environmental stress mechanisms. In this study, we identified and functionally characterized the BrPHD58 gene from *Brassica rapa*. Using sequence analysis, subcellular localization of BrPHD58–GFP fusion proteins, and transgenic *Arabidopsis thaliana* lines ectopically expressing BrPHD58, we investigated its role in salt stress responses, including seedling phenotypes and expression of salt-responsive genes. Subcellular localization analysis indicated that BrPHD58 predominantly accumulates within the nuclear compartment. Ectopic expression of BrPHD58 in *A. thaliana* significantly reduced seedling survival rates and root lengths under varying concentrations of NaCl compared to wild-type (WT) plants. Under soil-grown conditions, transgenic lines overexpressing BrPHD58 exhibited markedly decreased tolerance to salt stress. Moreover, ectopic expression of BrPHD58 led to a down regulation of key salt-responsive genes, *AtRD22, AtRD29A*, and *AtLEA14*, under salt stress conditions. Collectively, all these findings indicate that BrPHD58 acts as a negative modulator of salt stress tolerance in transgenic plants. Further investigation involving the development and analysis of BrPHD58 loss-of-function mutants in *B. rapa* is necessary to fully elucidate its physiological role in salinity adaptation.

## 1 Introduction

As sessile organisms, plants endure several detrimental environmental conditions, including elevated salt and drought stress, which significantly impact crop growth and yield and pose a serious threat to global food security (Van Velthuizen, 2007; Tebaldi and Lobell, 2018; Mittal et al., 2023). These stresses are anticipated to become more severe under ongoing climate change, further increasing yield losses in sensitive crop species. To mitigate these negative impacts, it is important to elucidate the complex regulatory components and signaling genes that govern the mechanisms of plant responses to salinity and drought stress (Hirayama and Shinozaki, 2010; Huang et al., 2012; Shimotohno et al., 2021).

The synthesis of downstream target genes is regulated by transcription factors (TFs), which are essential for controlling plant growth, developmental pathways, and abiotic stress responses (Udvardi et al., 2007; Bhoite et al., 2025). Zinc-finger proteins are a prominent class of TFs that enhance plant tolerance to various stress conditions. They are grouped into several classes based on the number and position of cysteine (C) and histidine (H) residues that coordinate different zinc-finger domains to exhibit zinc-binding activity. Examples include C2C2, C2H2, and C3H, which typically coordinate a single zinc ion, as well as C3HC4 RING finger, PHD (plant homeodomain), and LIM type domains, which coordinate two zinc ions (Takatsuji, 1999; Kosarev et al., 2002; Cassandri et al., 2017). The PHD finger was first described in Arabidopsis HAT3.1 and the maize homolog *ZmHOX1a* (Schindler et al., 1993). The PHD finger protein contains a Cys4HisCys3-type domain and has a structural similar to another RING finger domain, Cys3HisCys4-type, which harbors two zinc atoms in a cross-brace structure (Capili et al., 2001). Structurally, the PHD finger adopts a compact globular conformation characterized by a single α-helix and a two-stranded β-sheet (Quan et al., 2023). This structural feature allows the PHD finger to interact with a range of nuclear protein partners (Bienz, 2006; Mellor, 2006; Wysocka et al., 2006). Alfin1-like (AL) proteins constitute plant-specific PHD finger proteins, that were initially identified in alfalfa (*Medicago sativa* L.) as transcription factors (Winicov and Bastola, 1999). These proteins are characterized by the presence of an N-terminal Alfin/DUF3594 domain comprising approximately 130 amino acids and a C-terminal PHD domain comprising approximately 50 amino acid residues (Bastola et al., 1998). PHD finger domains, via ING and Alfin-like proteins, can recognize active histone marks, such as H3K4me2 and H3K4me3, suggesting their involvement in the chromatin-based regulation of gene expression (Lee et al., 2009). In addition to facilitating protein–protein interactions, the PHD domain appears to contribute to the nuclear localization of Alfin-like proteins (Wei et al., 2009). Many studies have shown that Alfin1-like PHD finger protein genes are involved in abiotic stress responses. Recent studies have demonstrated that overexpression of *AhAL1* enhances both salt and drought stress tolerances (Tao et al., 2018). In alfalfa, Alfin1 contributes to salt stress adaptation (Bastola et al., 1998; Winicov and Bastola, 1999), whereas in *Arabidopsis*, *AtAL5*, and in soybean, *GmPHD2* improves salt stress tolerance in transgenic plants (Wei et al., 2009, Wei et al., 2015). Additionally, *GmPHD5* mediates histone crosstalk between H3K4 methylation and H3K14 acetylation under salt stress, facilitating the recruitment of chromatin-remodeling complexes (Wu et al., 2011). Functional diversification has also been observed in the Arabidopsis AL protein family. For example, *AtAL6* functions in root hair elongation (Chandrika et al., 2013), whereas *AtAL7* acts as a negative regulator of salt stress tolerance in transgenic plants (Song et al., 2013). Similarly, *GhAL19* in cotton acts as a negative regulator of drought and salinity tolerance (Liu et al., 2024). Furthermore, *AtAL3* mutation moderately enhances salt stress tolerance in Arabidopsis transgenics (Song et al., 2013), implying that Alfin1-like PHD finger proteins may also participate in abiotic stress responses in Brassica species.

Brassica comprises economically important vegetable and oilseed crops, including the diploids *Brassica rapa* (AA, n = 10), *B. oleracea* (CC, n = 9), and *B. nigra* (BB, n = 8), and the amphidiploids *B. napus* (AACC, n = 19), *B. juncea* (AABB, n = 18), and *B. carinata* (BBCC, n = 17). These species are cultivated for a wide range of products, including vegetables, edible oils, and condiments (Ashraf and McNeilly, 2004). These vegetable crops are usually subjected to several environmental stresses that significantly influence crop yield. However, *B. rapa* is primarily a vegetable crop and is more sensitive to saline stress, especially during the early growing or seedling stages, where salinity strongly limits growth and physiological performance, including reduced germination, disrupted hormone responses, and inhibited root growth (Bray, 2000; Wang et al., 2023; Ma et al., 2025). In Brassica, salt tolerance is generally linked to ion homeostasis (Na^+^/K^+^ balance), osmotic adjustment, and ROS-scavenging capacity, which are regulated by hormone signaling and transcriptional or post-translational regulation (Wang et al., 2023; Ma et al., 2025). Previously, we identified 145 PHD finger genes in *B. rapa* and proposed their involvement in abiotic stress responses (Alam et al., 2019), motivating the continued functional analysis of this family of genes under salinity stress. These results indicate that *BrPHD58* is a nucleus-localized PHD finger protein that modulates salt stress responses in plants in a directionally specific manner. In this study, we cloned and characterized *BrPHD58* from *B. rapa* to determine its role in salt stress tolerance. We further assessed the impact of ectopic *BrPHD58* expression on seedling survival rate and root growth upon exposure to variable concentrations of NaCl and evaluated whole-plant salt tolerance in transgenic Arabidopsis. Together, these data suggest that *BrPHD58* acts as a negative regulator of salt stress responses in transgenic plants, providing a functional entry point to the AL subfamily in *B. rapa*, with implications for improving stress resilience.

## 2 Materials and methods

### 2.1 Plant growth conditions

Seeds of *B. rapa* (inbred line Chiifu-401-42) were germinated in Petri dishes at 25°C, transplanted to pots with fertile substrate, and grown at 22°C under a 16 h/8 h light–dark cycle. Seedlings were subjected to salinity stress via irrigation with 100 mM or 200 mM NaCl. Leaf and root tissues from control and treated plants were collected 12 h after treatment, snap-frozen in liquid nitrogen, and stored at −80°C for RNA extraction. Three biological replicates were used for each condition (OMEGA, China). Wild-type *Nicotiana benthamiana* was grown in a 2:1 (v/v) peat:perlite mix at 25°C, approximately 70% RH, under a 16 h/8 h photoperiod, and fully expanded leaves were used for Agrobacterium-mediated GFP transient expression.

### 2.2 Identification and sequence analysis

The full-length coding sequence (CDS) of the putative gene was retrieved from the *B. rapa* genome database (BRAD; http://brassicadb.org/brad/). The physicochemical properties of the identified proteins, including the theoretical isoelectric point (pI) and molecular weight (MW), were calculated using the ProtParam tool (Artimo et al., 2012). In addition, the *BrPHD58* protein sequence was used as a BLASTP query to identify *PHD58*-like homologs in *Arabidopsis thaliana* and other Brassica species by searching online proteomes in BRAD (http://brassicadb.org/brad/). The resulting candidate sequences were then confirmed through the presence of the conserved PHD-finger domain, together with the associated Alfin-like domain, using the SMART and InterPro databases. The exon/intron structure of the *BrPHD58* gene was determined using Gene Structure Display Server 2.0. Gene-specific primer pairs were designed using the Primer3Plus tool (https://www.primer3plus.com). High-quality total RNA was obtained from the young leaf tissue of *B. rapa* using an RNA purification kit (OMEGA, China), and 2 μg of high-quality RNA was subsequently used for first-strand cDNA synthesis using a cDNA Synthesis Kit (TransGen, China).

### 2.3 Evolutionary analysis

Evolutionarily conserved protein sequences from various Brassica species were identified through BLAST searches using the *B. rapa* genome database on the BRAD platform (http://brassicadb.org/brad/). Multiple sequence alignments were conducted using ClustalW, and ligand-binding residues were annotated with the use of BioEdit software (version 7.2.5). Phylogenetic relationships among the homologs were inferred using the neighbor-joining (NJ) method implemented in MEGA7 software (version 7.0.26), with statistical support evaluated through 1,000 bootstrap replicates.

### 2.4 Subcellular localization of *BrPHD58* protein

To investigate subcellular localization, the *BrPHD58* coding sequence, flanked by BamHI and XbaI restriction sites, was initially cloned into the pMD18-T cloning vector. The resulting recombinant plasmids were digested with the corresponding restriction enzymes and ligated into the 35S-GFP expression vector (35S-pBinGFP2) to generate the fusion construct 35S-BrPHD58-GFP. The integrity of the construct was confirmed by sequencing. The verified fusion plasmids were then transferred into *A. tumefaciens* strain GV3101 using standard transformation protocols. The fusion constructs and control vectors were introduced into the epidermal cells of *N. benthamiana* leaves via microinjection. The samples were then returned to the growth chamber for an additional 24 h, after which confocal microscopy was used to observe the transformed tobacco leaf cells (TCS sp8, Leica, Solms, Germany).

### 2.5 Target gene transformation

To amplify the target gene, PCR was performed using cDNA synthesized from total RNA as the template. The amplification fragments were separated using agarose gel electrophoresis and purified using a Gel Extraction Kit (OMEGA BioTek, USA). The purified DNA fragment obtained from gel extraction was subsequently ligated into the pMD-18T vector (TaKaRa, Dalian, China) for molecular cloning. The recombinant construct was digested using QuickCut restriction enzymes XbaI and BamHI (TaKaRa, Dalian, China). Following digestion, the products were separated on a 1% agarose gel, and DNA fragments of the expected size for the candidate gene were visualized and confirmed. The presence and accuracy of the target sequence were further verified using Sanger sequencing. The purified product was ligated into the expression vector pCAMBIA1301, driven by the CaMV35S promoter. The fusion plasmids were first validated by restriction enzyme digestion and subsequently confirmed by Sanger sequencing. After the successful insertion of the candidate gene into the 35S-pCAMBIA1301 vector, the recombinant construct was introduced into *Agrobacterium tumefaciens* strain GV3101 for expression studies.

### 2.6 Generation of *BrPHD58*-transgenic plants and NaCl stress conditions

Various Arabidopsis transgenic lines were established via *A. tumefaciens*-mediated transformation using the floral dip method (Mara et al., 2010). Putative T1 transgenic lines were validated by PCR amplification of *BrPHD58* from genomic DNA and RT-PCR analysis of RNA using specific primers (**Supplementary Table S1**). Total RNA was extracted and reverse-transcribed into cDNA according to the protocol provided by TransGen Bio-Tech, China. Genomic DNA was purified from the leaf tissue of transgenic Arabidopsis using the CTAB extraction technique. Seeds from confirmed T1 Arabidopsis lines were sown on MS medium containing 30 mg/L hygromycin to select for T2 and, subsequently T3 generations. Three homozygous T3 lines (2, 3, and 6) were selected for subsequent molecular and phenotypic characterization.

For the salt stress experiment, transgenic seeds were grown on half-strength MS medium. For the initial 5-day growth screen, seedlings were grown on half-strength MS medium with different NaCl concentrations (75 mM and 150 mM), and their phenotypic responses were recorded. For soil-based salinity experiments, 14-day seedlings were cultivated in soil and exposed to 200 mM NaCl solution to simulate salt stress conditions. Photographs were captured at various time points after exposure to salinity stress. To assess chlorophyll levels, 12-day seedlings of both transgenic and WT lines were treated with 200 mM NaCl for three days. Subsequently, 0.05 g of rosette leaves were harvested and homogenized in 3 mL of 80% acetone, and the extracts were centrifuged at 8,000 rpm for 5 min. After centrifugation, the clear supernatant was carefully decanted, and the absorbance was measured at 645 nm and 663 nm. The total chlorophyll content was quantified using the method described by Arnon (1949).

### 2.7 Expression of stress-responsive genes in *BrPHD58* transgenic plants

The qRT-PCR method was employed to quantify the expression of selected reference genes in *BrPHD58* transgenic and WT (Col-0) plants under normal conditions and after exposure to salt stress using gene-specific primers (**Supplementary Table S1**). Twelve-day-old Arabidopsis seedlings cultivated on treated MS medium were subjected to salt stress via foliar application of 100 mM NaCl solution for 2 h. Total RNA was extracted from both transgenic lines and Col-0 plants using a Plant RNA Extraction Kit (OMEGA, China). First-strand cDNA synthesis was performed using the EasyScript cDNA Synthesis Kit (TransGen, China). qRT-PCR was conducted using the ABI 7500 Fast RT-PCR System (Applied Biosystems, USA) according to the manufacturer’s protocol. Each biological sample was analyzed in triplicate to obtain average Ct values, and relative gene expression levels were quantified using the 2⁻ΔΔCt method (Schmittgen and Livak, 2008). Relative expression levels of genes were normalized using Arabidopsis Actin-2 as the internal control gene.

### 2.8 Statistical analysis

Data are presented as mean ± standard error (SE) of three biological replicates. Statistical significance was evaluated using Student’s t-test (*P <0.05 and **P <0.01) in IBM SPSS Statistics v22.

## 3 Results

### 3.1 Identification and functional analysis of the candidate gene *BrPHD58* in *B. rapa*

**Figure 1:**
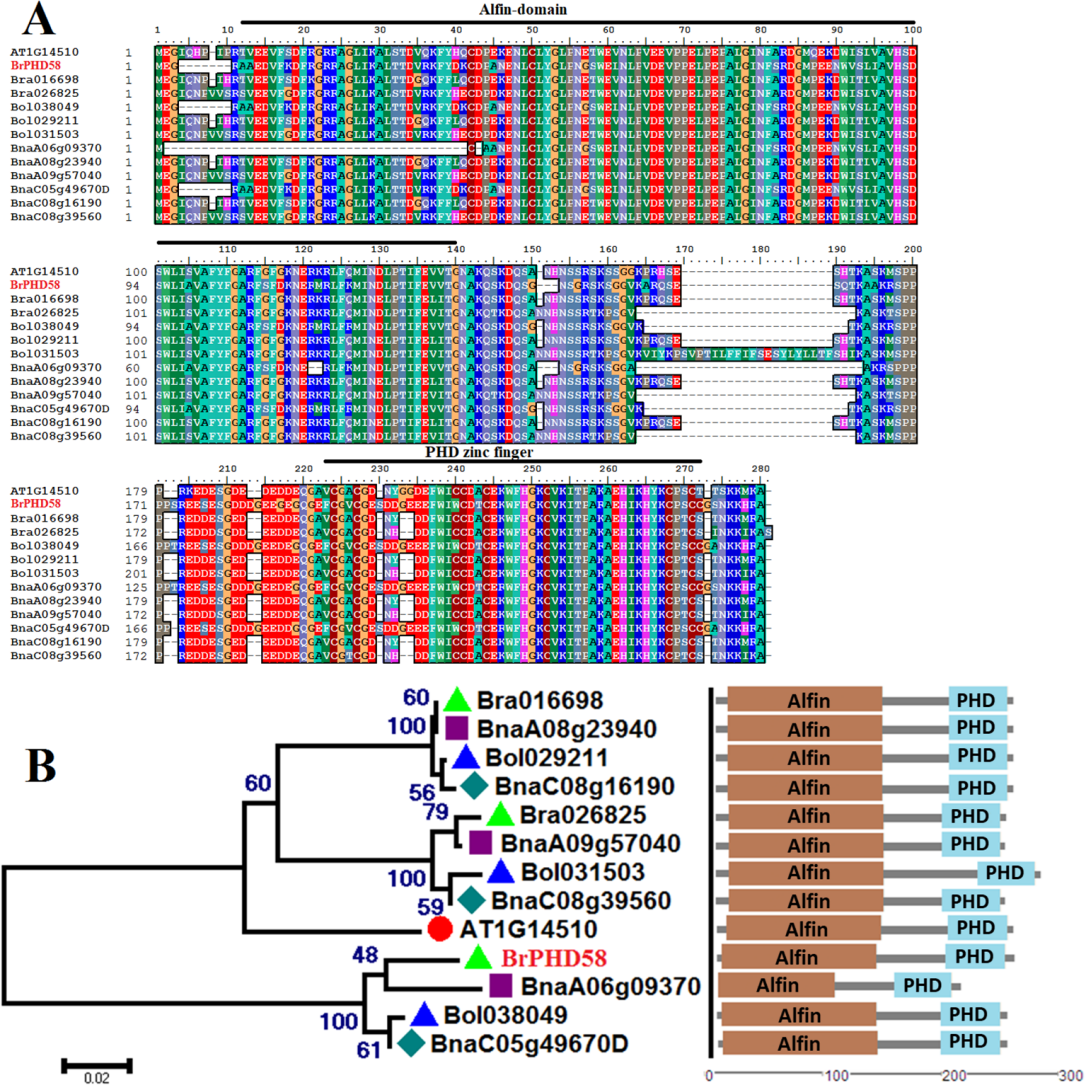
Multiple sequence alignment and phylogenetic analysis. **(A)** Amino acid sequences of *BrPHD58* and its closely related homologs from various Brassica species were aligned to examine sequence conservation. **(B)** A phylogenetic tree was created using NJ method implemented in MEGA7 software, preceded by the Alfin like PHD protein structure, including Alfin domain and PHD finger domain, where the Alfin domain and the PHD finger domain are indicated by brown and light-blue boxes, respectively. Bootstrap values based on 1,000 replicates are indicated at each node of the phylogenetic tree. Species abbreviations are as follows: At, *A*. *thaliana*; Bra, *B*. *rapa*; Bol, *B*. *oleracea*; Bn, *B*, *napus*.

The coding sequence of *BrPHD58* contained 753 bp, encoding a peptide sequence of 251 amino acids, with a predicted molecular weight of 27.91 kDa and an isoelectric point (pI) of 5.12. The *BrPHD58* gene is located on chromosome A06 and comprises five exons, interrupted by four introns. The SMART tool was used to analyze conserved domains and identified a canonical PHD finger domain of approximately 43 amino acids, typically implicated in chromatin recognition via histone methyl-lysine binding, and an Alfin-like region of approximately 128 amino acids, often associated with transcriptional regulation in plants (**Figure 1A**). BLASTP searches identified the closest homologs in *A. thaliana* and detected highly similar proteins across Brassica species (**Figure 1A**). A phylogenetic tree was established using the (NJ) method to evaluate the evolutionary association of Alfin-like PHD finger proteins among Brassica and Arabidopsis species. Phylogenetic analysis revealed that *BrPHD58* is closely related to other Brassica species (**Figure 1B**). Furthermore, conserved domain analysis revealed that proteins from all Brassica species contained a highly conserved N-terminal Alfin/DUF3594 domain and a C-terminal PHD domain, confirming their classification within the Alfin-like PHD-finger gene family (**Figure 1B**).

### 3.2 Expression analysis of *BrPHD58* under salt stress condition

Salt stress, drought, and temperature extremes are major abiotic factors that severely limit crop growth and lead to substantial yield loss (Gupta et al., 2022). In a previous study, we identified that *BrPHD58* was highly expressed under salt and drought stress conditions (Alam et al., 2019). Expression analyses were further performed in *B. rapa* using qRT-PCR to verify induction of *BrPHD58* by high salinity. The results clearly showed that *BrPHD58* was highly upregulated (e.g., 5-fold increase) at 12 h after treatment with 100 mM and 200 mM NaCl compared to the control (**Supplementary Figure S1**). These results suggest that *BrPHD58* may contribute to the regulation of plant responses to salt stress.

### 3.3 Functional characterization of *BrPHD58*

The *BrPHD58* gene (Bra026210), which encodes a PHD finger protein in *B. rapa*, was amplified using gene-specific primers (**Supplementary Figure S2A**). The PCR product was purified, ligated into the pMD18-T vector, and transformed into *E. coli* DH5α. Recombinant clones were confirmed by Sanger sequencing and double enzyme digestion, producing two expected bands of approximately 2,700 bp and 753 bp (**Supplementary Figures S2B, D**). For expression analysis, the *BrPHD58* fragment was ligated into the 35S-GFP and 35S-pCAMBIA1301 vectors. Precise insertion into 35S-GFP was confirmed by sequencing and BamHI/XbaI digestion (**Supplementary Figure S2C**). Similarly, cloning into 35S-pCAMBIA1301 was validated by sequencing and XbaI/BamHI digestion, producing bands of approximately 12 kb and 753 bp, respectively (**Supplementary Figure S2E**). The confirmed constructs were introduced into *A. tumefaciens* GV3101 for downstream expression analysis. Six transgenic lines were further confirmed by genomic PCR and used for subsequent *BrPHD58* expression analyses (**Supplementary Figures S2F, G**).

### 3.4 Subcellular localization of *BrPHD58*

**Figure 2:**
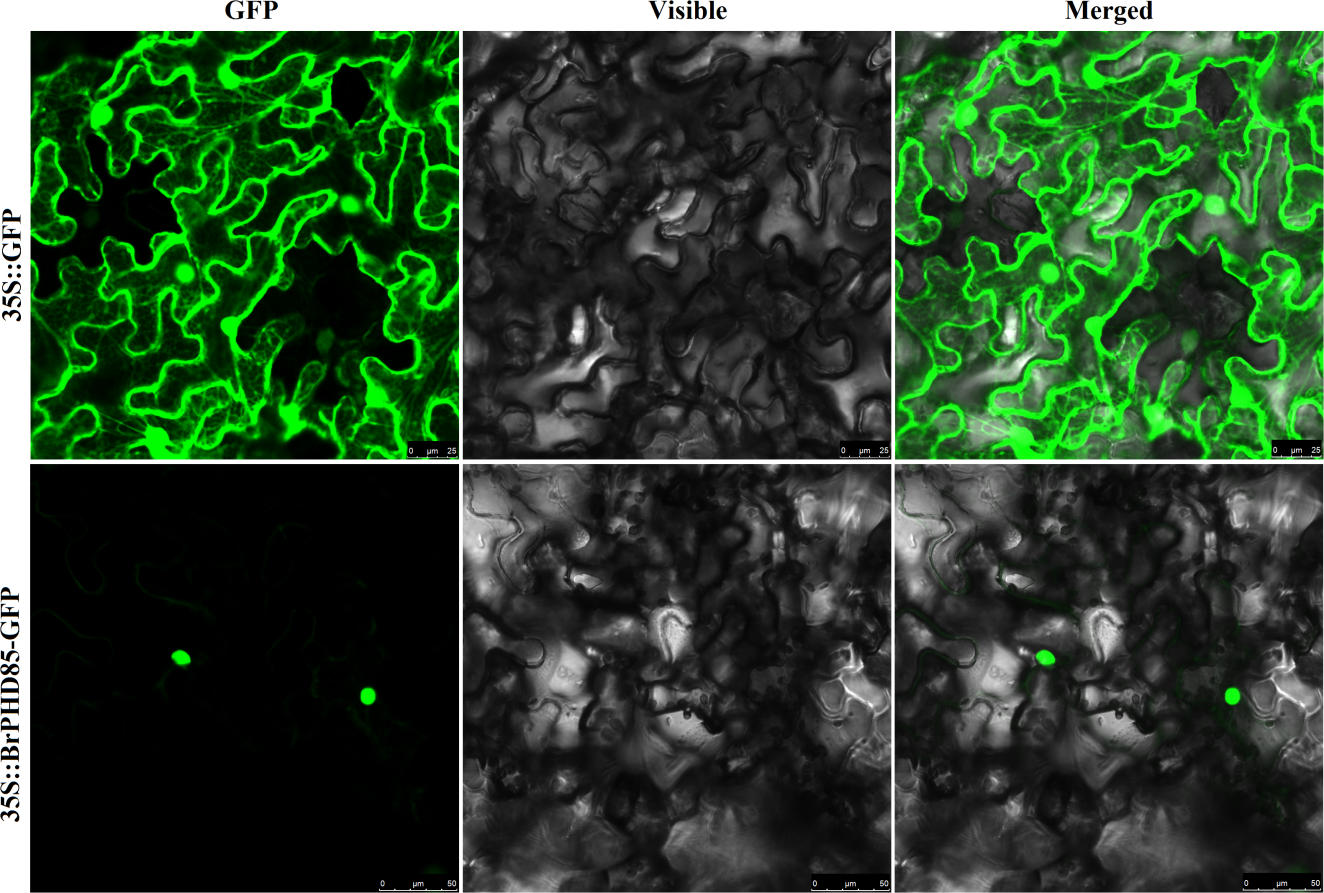
Subcellular localization of *BrPHD58*-GFP fusion proteins in *N. benthamiana.* Confocal scanning microscopy was employed to examine the intracellular localization patterns of both the control GFP and the *BrPHD58*-GFP fusion proteins expressed in *N. benthamiana* leaves.

To investigate the subcellular localization of *BrPHD58*, a BrPHD58-GFP fusion construct under the 35S promoter was transiently expressed in *N. benthamiana* leaves via *A. tumefaciens*-mediated transformation. The 35S:GFP vector was used as a control. GFP fluorescence was visualized using confocal microscopy. The results confirmed that the *BrPHD58* protein was specifically localized in the nucleus, as indicated by the distinct nuclear GFP fluorescence observed, whereas the control GFP signal was distributed across the cell (**Figure 2**). These findings suggest that *BrPHD58* predominantly resides in the nucleus, supporting its potential role as a transcription factor (**Figure 2**).

### 3.5 Ectopic expression of *BrPHD58* under salt stress condition

**Figure 3:**
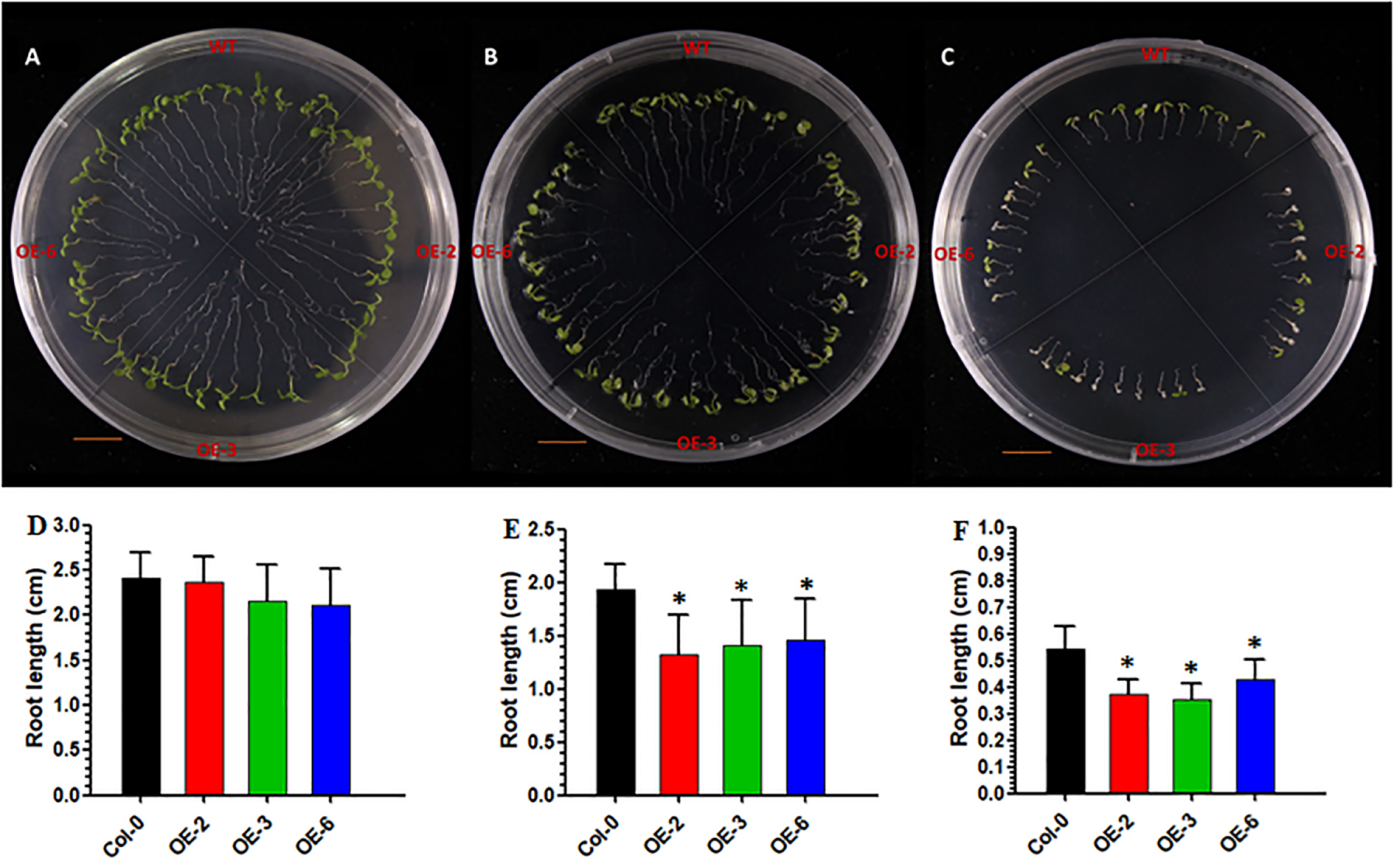
Survival rates and root lengths of *BrPHD58* transgenic seedlings at different NaCl concentrations. **(A, D)** Seedlings grown on one-half MS medium (control); **(B, E)** Seedlings cultured on one-half MS medium supplied with 75 mM NaCl; **(C, F)** Seedlings cultured on one-half MS medium supplied with 150 mM NaCl. Root length was recorded following five days of treatment. The x-axis shows the WT (Col-0) and transgenic lines, whereas the y-axis indicates root length (cm) **(D–F)**. Statistical comparisons were performed employing Student’s t-test, with significance levels determined as *P <0.05 relative to Col-0 control plants.

To assess the physiological responses to salt stress, three independently derived homozygous *BrPHD58* (Bra026210) overexpression lines were selected for a comprehensive analysis based on their high expression levels as determined by RT–qPCR (**Supplementary Figure S3**). Under standard one-half MS growth conditions, the growth performance of the three transgenic lines was largely comparable to that of WT (Col-0), with only minimal differences observed (**Figures 3A, D**). However, upon exposure to salt stress, the growth of all *BrPHD58* transgenic lines was significantly affected compared to that of WT (Col-0) (**Figures 3B, C**). Moreover, the *BrPHD58* transgenic lines exhibited a significant reduction in root length compared to the WT control under 75 mM and 150 mM NaCl treatment (**Figures 3E, F**). These findings suggest that ectopic expression of *BrPHD58* adversely affects root development under salt stress and reduces the overall salt tolerance of *A. thaliana* seedlings, underscoring its potential role in the modulation of stress response mechanisms.

### 3.6 Overexpression of *BrPHD58* negatively modulates the salt stress response in Arabidopsis

**Figure 4:**
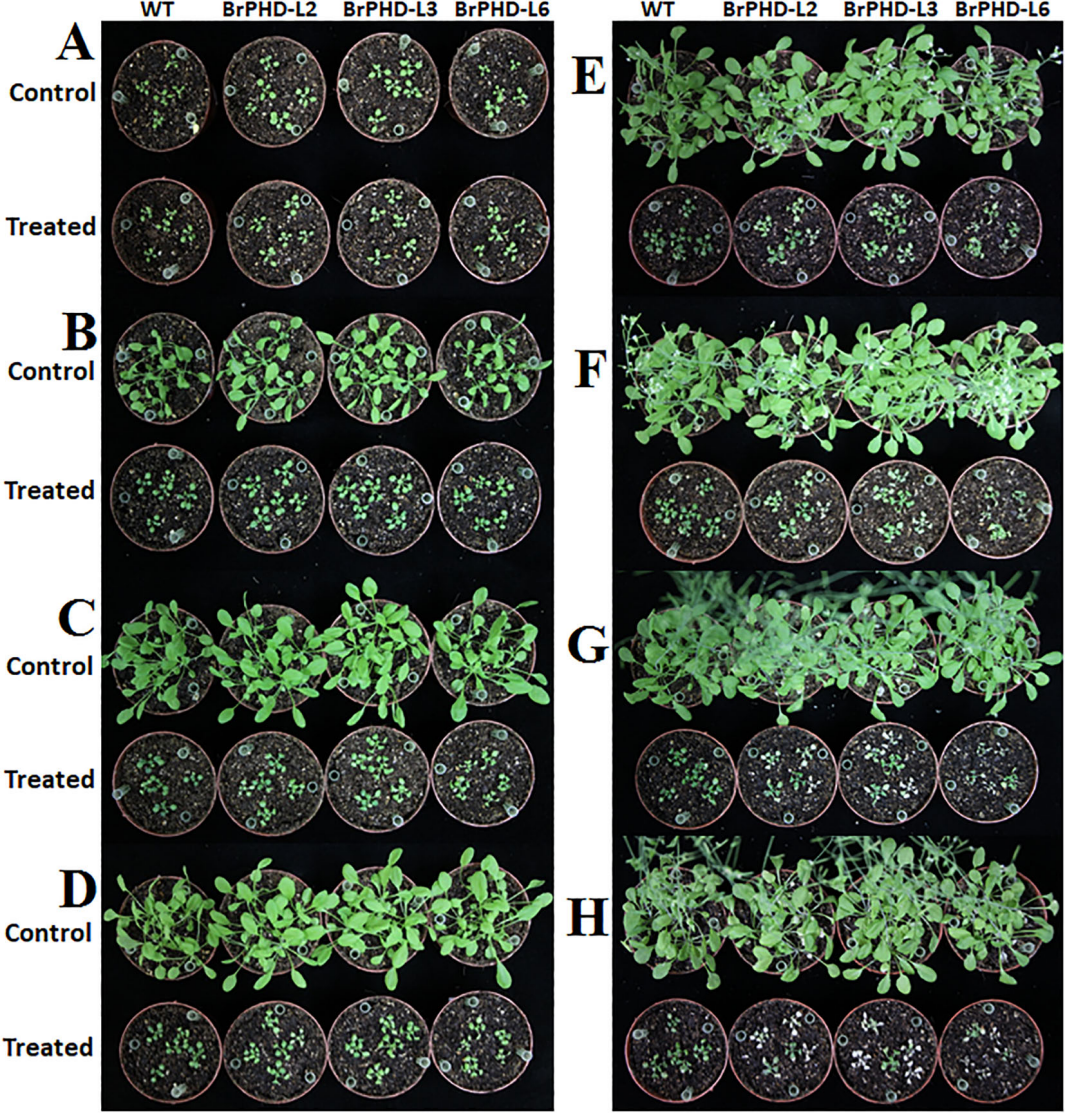
Overexpression of *BrPHD58* in Arabidopsis reduces tolerance to salt stress. Fourteen days seedlings of WT (Col-0) and *BrPHD58* T3 transgenic lines were treated with 200 mM NaCl treatment to assess their response to salt stress. **(A)** Phenotypes of (WT) Col-0 and *BrPHD58* OE lines under normal growth conditions. **(B)** Plant responses after 7 days of treated with salt stress **(C)** Phenotypes following 14 days of salt stress. **(D)** Phenotypes after 18 days of salt exposure. **(E)** Responses observed after 21 days under salt stress. **(F)** Phenotypes following 24 days of salt exposure. **(G)** Phenotypes after 27 days of salt stress. **(H)** Phenotypes after 30 days of salt stress exposure.

**Figure 5:**
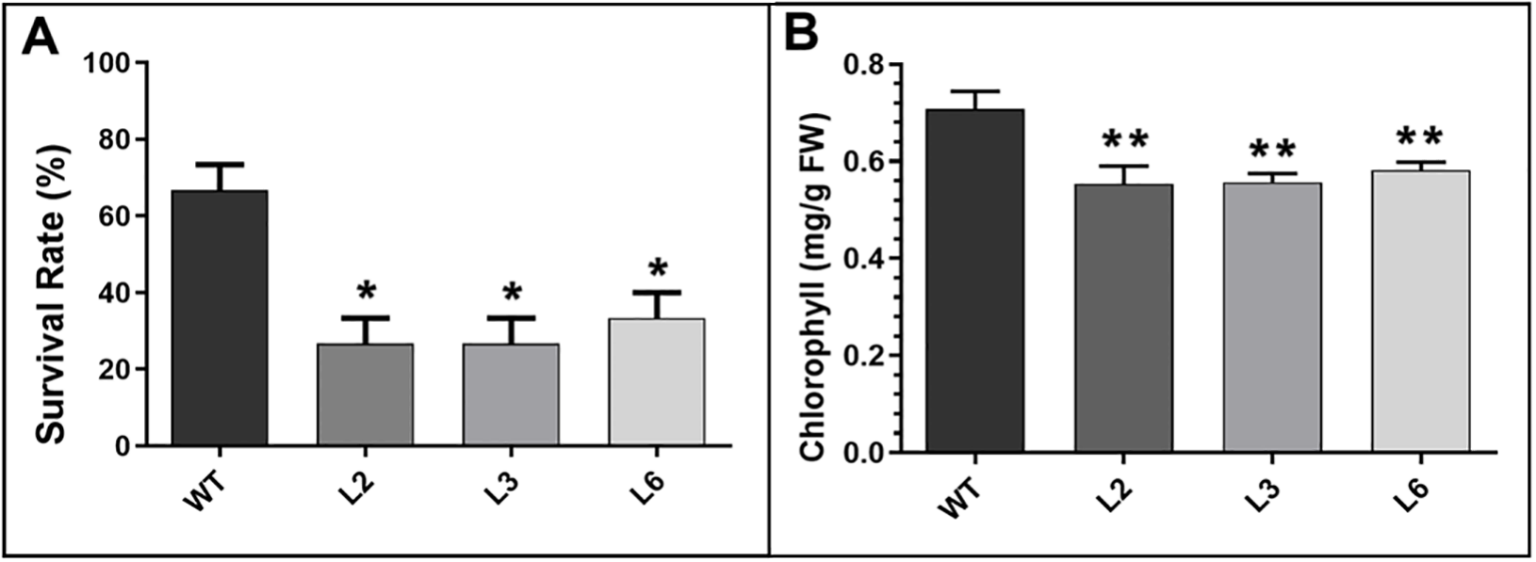
Evaluation of phenotypic responses in *BrPHD58*-overexpressing Arabidopsis lines subjected to high salinity conditions. **(A)** Survival rates of WT and *BrPHD58*-OE plants following 21 days of exposure to salt stress. **(B)** Chlorophyll content was assessed in *BrPHD58*-OE Arabidopsis lines and WT Col-0 seedlings. Measurements were taken after 12-day plants were exposed to 200 mM NaCl for three consecutive days. All experiments were conducted in triplicate. Student’s t-test was used for significant. Differences relative to WT Col-0 plants are showed by *P <0.05 and **P <0.01.

To investigate whether *BrPHD58* transcriptional factors have a functional role in response to salt stress, we developed transgenic Arabidopsis lines expressing *BrPHD58* driven by the constitutive CaMV 35S promoter. Three independent Arabidopsis transgenic lines (L2, L3, and L6) were cultivated in soil, and 14-day-old T3 seedlings were treated with 200 mM NaCl (**Figure 4**). Overexpression of *BrPHD58* impaired salt stress tolerance in Arabidopsis, as evidenced by the phenotypic differences observed after 7 days of 200 mM NaCl treatment (**Figure 4A**). Transgenic lines displayed leaf chlorosis compared to the Col-0 controls (**Figure 4B**). After 14 days, some of the transgenic plants died, whereas the majority of Col-0 plants remained green and viable (**Figure 4C**). By 21 days, most of the transgenic lines had died (**Figure 4E**), and after 27 days almost all of the transgenic lines had wilted and died, whereas some of the Col-0 plants survived at 200 mM NaCl (**Figure 4G**). These results indicate that *BrPHD58* overexpression reduces salt tolerance in Arabidopsis. Survival rates recorded across the three independent salt stress experiments revealed significant differences between the transgenic lines and WT (Col-0) plants (**Figure 5A**). Chlorophyll content was measured in 12-day-old seedlings of various transgenic and WT plants following a 3-day treatment with 200 mM NaCl, revealing significant differences between the two groups (**Figure 5B**). The results demonstrated a significant reduction in chlorophyll levels in the transgenic lines compared to the controls (**Figure 5B**). These findings imply that *BrPHD58* overexpression impairs the plant’s capacity to cope with salt stress.

### 3.7 Differential expression profiles of salt stress-related genes in *BrPHD58* transgenic Arabidopsis lines

**Figure 6:**
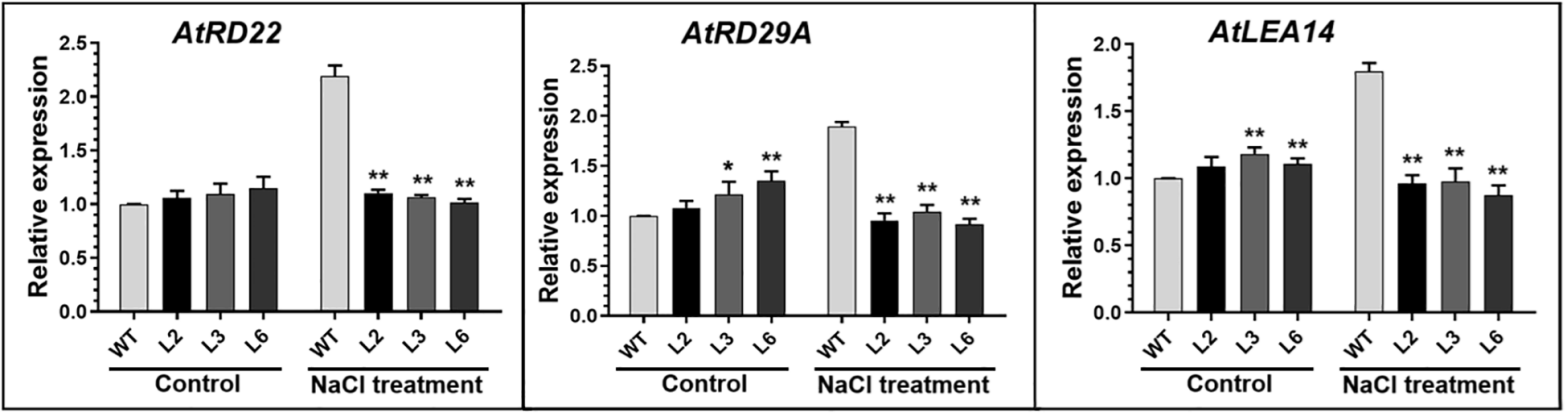
Expression levels of stress-related genes in *BrPHD58*-overexpression and WT plants. The expression levels of stress related genes were analyzed through qRT-PCR, with ACTIN2 serving as the internal reference. The x-axis represents the WT (Col-0) and transgenic lines, while the y-axis indicates relative expression levels. Statistical significance was shown as *P <0.05 and **P <0.01.

To explore the molecular mechanism of *BrPHD58* in the salt stress response, we performed quantitative expression analysis of three stress-associated genes in both *BrPHD58* transgenic lines and Col-0 plants. The results demonstrated that under normal conditions, the transcript levels of *AtRD22*, *AtRD29A*, and *AtLEA14* were elevated in *BrPHD58* plants compared to those in WT. However, following salt stress treatment, the expression levels of the stress-responsive genes *AtRD22*, *AtRD29A*, and *AtLEA14* were significantly reduced in *BrPHD58* transgenic plants compared to WT plants (**Figure 6**). These results suggest that *BrPHD58* may function as a negative regulator of gene expression associated with salt stress response in plants.

## 4 Discussion

Zinc finger proteins are widespread in plants and often function as transcriptional regulators of developmental and stress responses (Takatsuji, 1999). PHD finger proteins constitute a prominent class of transcriptional regulators in plants (Quan et al., 2023) and have been increasingly implicated in abiotic stress adaptation, including responses to salinity and drought (Winicov and Bastola, 1999; Lee et al., 2009; Wei et al., 2009, Wei et al., 2015; Tao et al., 2018). Consistent with this, we previously identified 145 PHD finger genes in *B. rapa* and observed stress-responsive expression patterns, suggesting their potential roles in abiotic stress adaptation (Alam et al., 2019). Based on these findings, we cloned *BrPHD58*, an Alfin-like PHD finger transcription factor from *B. rap*a, to investigate its functional role in the plant response to salt stress. Phylogenetic analysis revealed that *BrPHD58* shares a high degree of sequence similarity with Arabidopsis *AtAL7*. Notably, previous research has shown that *AtAL7* acts as a negative regulator of salt stress tolerance in transgenic plants (Song et al., 2013). Many studies have proposed that within a gene family, different members contain variant expression patterns and subcellular localizations for different functions (Zhang et al., 2014; Yao et al., 2015). The ability of Alfin-like PHD finger proteins to bind to the histone markers H3K4me3/2 and cis-element GNGGTG/GTGGNG indicates their function as transcriptional regulators. Several other studies have shown that Alfin-like PHD finger proteins are localized in the nucleus (Wei et al., 2009, Wei et al., 2015). In the present study, *BrPHD58* was localized to the nucleus (**Figure 2**), consistent with many transcriptional regulators that function in the nucleus to control gene transcription and expression and contribute to functional diversification within a gene family, such as the Alfin-like PHD finger family (Yao et al., 2015; Zhang et al., 2016; Yang et al., 2017). Functional studies of Alfin genes across various plant species, such as *Alfin1* in alfalfa, *AhAL1* in *Atriplex hortensis*, *GmPHD2* in soybean, and *AtAL5* in *A. thaliana*, have demonstrated that these genes enhance stress tolerance when overexpressed in transgenic plants (Bastola et al., 1998; Winicov and Bastola, 1999; Wei et al., 2009, Wei et al., 2015; Tao et al., 2018). In contrast, overexpression of *AtAL7* in Arabidopsis resulted in reduced salt stress tolerance, whereas loss-of-function mutants of *AtAL7* exhibited increased root length under salt stress conditions, implying that *AtAL7* functions as a negative regulator of salt tolerance mechanisms (Song et al., 2013). In Arabidopsis, *AtAL3* and *AtAL7* have been shown to negatively affect salt stress tolerance, whereas *AtAL5* enhances it (Song et al., 2013; Wei et al., 2015). Similarly, in *A. hortensis*, *AhAL1* has been shown to improve salt stress resistance in transgenic Arabidopsis, whereas the remaining three *AhAL* genes induce hypersensitivity to salt stress (Tao et al., 2018). This different behavior suggests functional divergence within the gene family, potentially driven by distinct chromatin-remodeling environments, differential cis-regulatory elements, or lineage-specific adaptive pressures that modulate their transcriptional and post-transcriptional responses under stress. In *Populus trichocarpa*, five of the nine *PtAL* genes showed a slight upregulation in expression with prolonged stress exposure. Notably, *PtAL4* and *PtAL6* exhibited downregulated expression following drought and salt treatments, whereas the remaining PtAL genes displayed only marginal changes compared to the control conditions (Sabir et al., 2023). In another case, the mutation of *AtAL3* in *Arabidopsis* moderately increased salt tolerance in transgenic plants (Song et al., 2013). However, this study demonstrated that *BrPHD58* transgenic lines exhibited reduced root length compared to controls when exposed to 75 mM and 150 mM NaCl (**Figures 3A–F**), consistent with observations reported in previous studies involving transgenic plants. Phenotypic assessments and survival rate analyses revealed that ectopic overexpression of *BrPHD58* diminished salt stress tolerance in transgenic plants (**Figures 4A–H**). Similar phenotypic phenomena have also been reported in several earlier studies; for example, the overexpression of *GhWRKY17* markedly reduced drought and salt stress tolerance in transgenic tobacco plants (Yan et al., 2014), and *GmWRKY13* led to sensitivity to abiotic stresses (Zhou et al., 2008). These results are consistent with previous findings regarding *AtAL7* and *PtAL4/6* (Wei et al., 2015; Sabir et al., 2023), underscoring the important role of the Alfin-like (AL) gene family in regulating growth and stress tolerance in Brassica and other plants. Additionally, the expression of *AtWRKY15* has been shown to increase sensitivity to salt and oxidative stress (Vanderauwera et al., 2012), while overexpression of *ZmWRKY17* similarly heightened salt stress sensitivity in Arabidopsis (Cai et al., 2017). Chlorophyll content is an important indicator of photosynthetic efficiency and overall plant growth. Specifically, one of the most important roles of these functions is the transfer of light energy (Rong et al., 2025). In the present study, the total chlorophyll content of the transgenic lines was reduced compared to that of the control, indicating that *BrPHD58* adversely influenced photosynthetic activity (**Figure 5B**). Similar results were obtained in another study in which overexpression of *WRKY75* reduced salt tolerance by promoting reactive oxygen species (ROS) accumulation in both *A. thaliana* and *B. napus.* Furthermore, the chlorophyll content of Col-0 was higher than that of the transgenic lines (Ping et al., 2024). Additionally, under salt stress conditions, *GmPHD5* mediates the interaction between methylated H3K4 and acetylated H3K14, potentially facilitating the recruitment of chromatin remodeling complexes and transcription factors responsible for managing the expression of stress-responsive genes, including *GmRD22* and *GmGST* (Wu et al., 2011). Similarly, a group of *LEA* genes, known for their critical roles in stress tolerance, has been extensively investigated in the context of plant responses to various abiotic stresses (Liu et al., 2019; Wang et al., 2019). Recently, it was demonstrated that cotton *GhAL19* acts as a negative regulator of salt and drought tolerance by modulating antioxidant activity and the ABA-mediated signaling pathway (Liu et al., 2024). In addition, the transcript level of *LEA14* gene was upregulated in knockdown lines of *GhAL19* under salt and drought stress (Liu et al., 2024). Consistent with these findings, our study showed that the expression levels of stress-associated genes were significantly reduced in salt-treated transgenic lines compared to the WT controls, whereas under non-stress conditions, these genes exhibited similar or elevated expression levels (**Figure 6**). Overall, these results indicate that *BrPHD58* acts as a negative regulator in salt stress environments. Consequently, further investigation is required to elucidate the underlying molecular mechanisms and regulatory pathways involved in salt stress conditions.

## 5 Conclusion

In conclusion, we successfully cloned and characterized *BrPHD58* from *B. rapa*. A subcellular localization study revealed that *BrPHD58* is specifically localized in the nucleus, implying its involvement in nuclear regulatory mechanisms potentially related to stress response pathways. Functional analysis in Arabidopsis further showed that *BrPHD58* overexpression significantly decreased seedling survival and inhibited root growth under salt stress, indicating that *BrPHD58* negatively regulates salt tolerance by modulating the expression of stress-responsive genes. These results advance our understanding of Alfin-like PHD-finger proteins by associating *BrPHD58* with a measurable stress-responsive phenotype and highlight this regulatory factor as a potential target for manipulating abiotic stress responses in *B. rapa*. However, the precise molecular basis of *BrPHD58* function remains unclear. Future studies should identify its downstream targets and interacting partners and validate its function in *B. rapa* loss-of-function backgrounds under salt stress conditions.

## Data Availability

The original contributions presented in the study are included in the article/**Supplementary Material**. Further inquiries can be directed to the corresponding authors.
