# Supplementary material for: Functional characterization of BrPHD58, an Alfin-like PHD finger protein from Brassica rapa, reveals its negative role in salt stress tolerance in Arabidopsis thaliana
Source: Front Plant Sci. 2026 Jan 28;17:1749944. doi: 10.3389/fpls.2026.1749944 (PMC12892343; doi:10.3389/fpls.2026.1749944)
Supplement: Supplementary file 1 [file DataSheet1.zip › Data Sheet 1.pdf]

Table S1. The information of primers used in current studies

No	Gene Name	Sequence of Primers (5'-3')*
1	*BrPHD58*	F: CATGTCTAGAATGGAGGGACGCGCGGCT
		R: CCCGGATCCTCAAGCTCTGTGTTTCTTGT
2	Subcellular localizatin of *BrPHD58*	F: CATGGGATCCATGGAGGGACGCGCGGCT
		R: CCCTCTAGAATCAAGCTCTGTGTTTCTTGT
3	qPCR of *BrPHD58*	F: GGTGAGTTTTGTGGGGTCTG
		R: AGCTCTGTGTTTCTTGTTGG
4	*BrActin*	F: TCAGATGCCCAGAAGTCTTGTTCC
		R: TCCTCCACCTGCCTCATCATACTC
5	*AtRD22* (At5G25610)	F: GCTGGGGTAAAGAAGTTGTC
		R: TTCCAAGCTGAGGTGTTCTT
6	*AtRD29A* (At5G52310)	F: CCCACCAAAGAAGAAACTGGAG
		R: GGCGAATCCTTACCGAGAACAG
7	*AtLEA14* (AT1G01470)	F:GATTTCTTCTGATCGACAAAACCTA
		R:AGCAAACCCAACTTATTACATTACG
8	*ACT2* (AT3G18780)	F: ACATTGTGCTCAGTGGTGGA
		R: TCATACTCGGCCTTGGAGAT
_	* F, farward primer; R, reverse primer.	

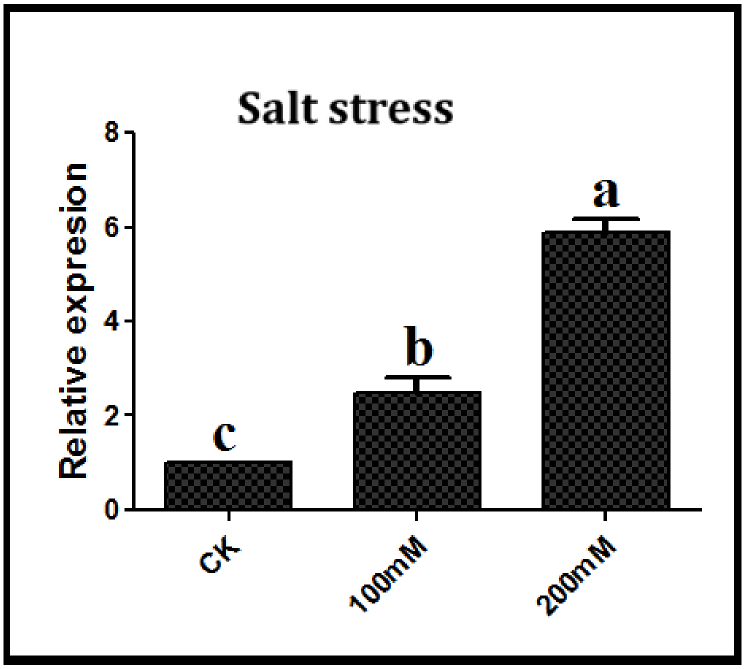

Figure S1. Relative expression patterns of *BrPHD58* gene under salt treatment by RT-qPCR. The different concentration represent by x-axis and the scale of relative expression showed by y-axis. Tukey tests were used to determine differences among effects on different time courses under salt treatment and different letters indicate significant difference ($p < 0.05$).

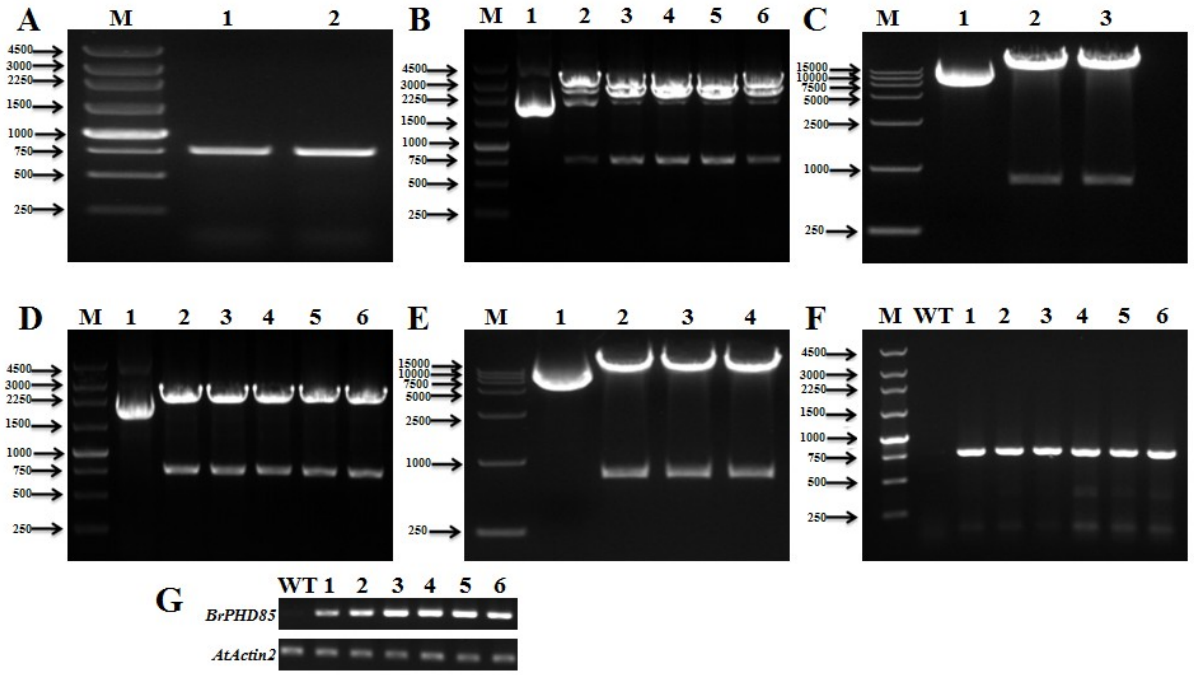

Figure S2. PCR Amplification of BrPHD58 and confirmation through enzyme digestion. A) M: 250bp DNA marker; Lane1-2: 753bp amplified fragment, B) M: 250bp DNA marker; Lane2: Recombinant pMD18T plasmid; Lane2-6: double enzyme digestion, C) M: DL15000bp DNA marker; Lane1: Recombinant 35S-GFP-plasmid; Lane2-3: double enzyme digestion, D) M: 250bp DNA marker; Lane1: Recombinant pMD18T plasmid; Lane2-6: double enzyme digestion, E) M: DL15000bp DNA marker; Lane1: Recombinant 35S-pCAMBIA1301 plasmid; Lane 2-4: double enzyme digestion, F) M: 250bp DNA marker; WT: control plant(Col-0); Lane 1-6: transgenic plants amplification, G) WT: control plant (Col-0) and Lane 1-6: transgenic plants expression of *BrPHD58* and Arabidopsis Actin2.

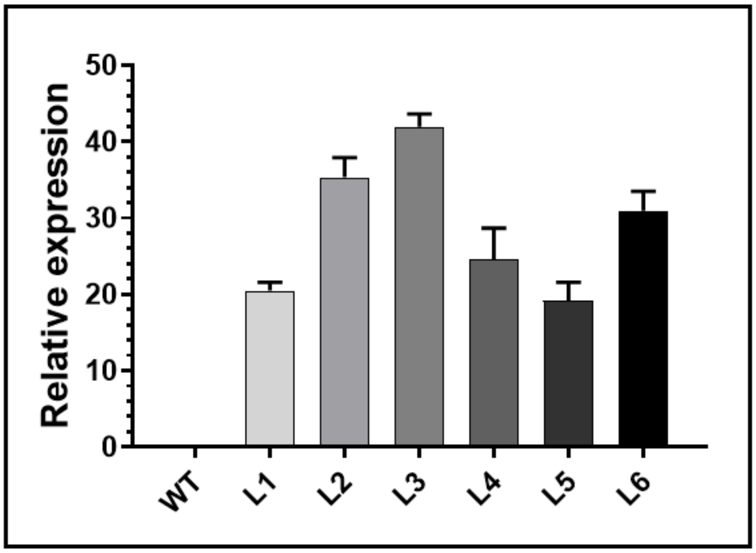

Figure S3. The relative expression of *BrPHD58* in transgenic Arabidopsis lines. The different overexpression lines and WT are shown on the x-axis and the relative expression level is shown on the y-axis.
